# Serum thymidine kinase 1 levels predict cancer-free survival following neoadjuvant, surgical and adjuvant treatment of patients with locally advanced breast cancer

**DOI:** 10.3892/mco.2013.149

**Published:** 2013-07-19

**Authors:** FEIYU CHEN, LILI TANG, TING XIA, ELLEN HE, GUOZHU HU, YUAN LI, MING ZHANG, JI ZHOU, STAFFAN ERIKSSON, SVEN SKOG

**Affiliations:** 1 Department of Breast Surgery, Xiangya Hospital, Central South University, Changsha, Hunan, P.R. China; 2 Department of Breast Oncology, The Second Affiliated Hospital of Guangzhou Medical College, Guangzhou, P.R. China; 3 Sino-Swedish Molecular Bio-Medicine Research Institute, Shenzhen, Guangdong, P.R. China; 4 Institute of Clinical Medicine, Jiangxi Provincial People’s Hospital, Nanchang, Jiangxi, P.R. China; 5 Department of Anatomy, Physiology and Biochemistry, Swedish University of Agricultural Sciences, Uppsala, Uppland, Sweden

**Keywords:** thymidine kinase 1, serum, breast cancer, survival

## Abstract

In this study, the use of serum thymidine kinase 1 protein (STK1p) concentration for the prognosis of the overall survival of patients with locally advanced breast cancer (n=51) following routine treatment (neoadjuvant treatment, surgery and chemotherapy) was investigated. The patients were followed up for 44 months and the STK1p values were determined by a high-sensitivity enhanced chemiluminescence (ECL) dot blot assay. The variables investigated in relation to metastasis and survival were STK1p, clinical stage, tumor size and age, by the Kaplan-Meier method, the log-rank test and Cox uni- and multivariate analyses. Patients with high STK1p values (≥2.0 pM) 3–6 months after surgery exhibited a positive correlation to clinical stage, tumor size, occurrence of metastasis and survival. The hazard risk for the development of metastatic disease and mortality among breast cancer patients was 11–12 times higher in patients with high compared to those with low STK1p values (<2.0 pM). Notably, patients with stage III/IV disease and low STK1p values exhibited statistically significantly improved survival compared to patients with high STK1p values. A multivariate Cox analysis demonstrated that the STK1p levels 6 months after surgery was the only independent prognostic factor for metastasis and survival. In conclusion, STK1p is a prognostic marker in patients with locally advanced breast cancer and it may help identify a subgroup of stage III/IV patients with improved cancer-free survival expectancy, enabling personalized treatment.

## Introduction

The conventional treatment of patients with advanced breast cancer currently involves three steps: i) neoadjuvant chemotherapy; ii) surgery of different degrees and iii) adjuvant chemotherapy. The neoadjuvant and adjuvant chemotherapy comprise different drug combinations, according to the individual patient situation. Although the neoadjuvant therapy has not demonstrated convincing evidence regarding survival benefit (1–6), it has certain advantages, such as downstaging of an inoperable cancer to an operable one, increasing the possibility of breast-conserving surgery and potentially reducing the risk of metastatic disease (1,7). Although the type of therapy is important for survival and quality of life after treatment, the prognosis prior to treatment initiation and the monitoring of the outcome following treatment are equally important. Therefore, imaging in combination with biopsy are commonly used techniques and the benefit of these techniques may be further improved by the use of serum biomarkers.

Serum thymidine kinase 1 (STK1) levels have been used for monitoring tumor therapy and for assessing the risk of recurrence and prognosis of survival in hematological malignancies as well as in solid tumors (8). Thymidine kinase 1 (TK1), a marker of cell proliferation, is an enzyme involved in nucleotide metabolism and is important for the supply of thymidine monophosphate for DNA synthesis. TK1 is activated during the late G1 stage of the cell cycle, reaches a peak during the late S and G2 phase and is degraded during mitosis. There are two types of thymidine kinase (TK), cytoplasmic (TK1) and mitochondrial (TK2); however, the expression of the latter is not associated with cell proliferation (9,10). TK1 serum levels are determined by its activity (STK1a) or its concentration (STK1p) (8), which is determined by specific anti-TK1 antibodies (SSTK Biotech Ltd., Shenzhen, China). The STK1a assay is mainly used for patients with leukaemia and lymphoma, whereas STK1p is useful for hematological malignancies as well as solid tumors, such as breast carcinoma.

In this study the use of serum thymidine kinase 1 protein (STK1p) concentration for monitoring the results of treatment (neoadjuvant, surgical and adjuvant) in patients with locally advanced breast cancer was investigated, including determination of the risk of metastatic disease following completion of treatment and prognosis of overall survival.

## Patients and methods

### Study design

The purpose of this study was to investigate the use of STK1p in patients with locally advanced breast cancer for the prognosis of the treatment outcome. The main endpoints were STK1p in relation to the development of metastasis and to survival. Patients were administered neoadjuvant treatment for 1 month, followed by surgery and adjuvant chemotherapy for 6 months. Serum samples were collected following neoadjuvant treatment and prior to surgery, and at 1, 3 and 6 months following surgery. Healthy age-matched females were used as controls. The development of metastasis and survival were followed up for 44 months. The variables considered were tumor clinical stage, tumor size, age, clinical response, metastasis and survival. The number of specimens (n=51) was selected in order to obtain statistically significant results. The details are described below.

### Patients

A total of 51 female patients with early locally advanced (n=2), locally advanced (n=47) and advanced (n=2) breast cancer were recruited in accordance with the NCCN guidelines for neoadjuvant chemotherapy (11) at the Department of Breast Surgery, Xiangya Hospital, Changsha, China, between 2007 and 2011. The breast cancer patients were diagnosed using cutting needle biopsy and the tumor clinical stage and size were determined using imaging in combination with vernier calipers. The majority of the patients (n=49) had invasive ductal carcinoma, whereas the remaining 2 patients had early locally infiltrating ductal tumors. The pathological type of the tumors was assessed after neoadjuvant treatment. All the patients received standard treatment protocols, i.e., 1 cycle of neoadjuvant chemotherapy, followed by surgery and 2 cycles of adjuvant chemotherapy. The surgical treatment was mastectomy in all the cases.

Information regarding clinical stage, tumor size and STK1p levels were obtained from all the patients, whereas information regarding response to treatment [complete response (CR), partial response (PR), stable disease (SD) and progressive disease (PD)] was available for 43 patients and survival for 38 patients. The clinical response was assessed after the last cycle of neoadjuvant treatment, prior to surgery. Staging was performed according to the International Union Against Cancer TNM staging system (12). The clinical stage was based on imaging and cutting needle biopsy techniques and the tumor size on imaging, all performed prior to neoadjuvant treatment. According to the AJCC guidelines for breast cancer staging, the primary tumors were classified into two groups: <5.0 and ≥5.0 cm, based on the greatest dimension, roughly corresponding to stage I/II and III/IV disease, respectively. Estrogen receptor (ER), progesterone receptor (PgR) and human epidermal growth factor receptor 2 (HER2) were determined after surgery using immunohistochemistry. Serum samples were collected 1 week after the completion of the neoadjuvant treatment and prior to surgery, and at 1, 3 and 6 months after surgery. Thus, the serum samples following surgery were collected during the adjuvant chemotherapy period. The characteristics of the patients are presented in Table I. The mean age of the patients was 45.9±9.3 years (range, 30–67 years). Age-matched healthy females (n=286), without any tumor, infection, or other non-tumor diseases or symptoms, were used as controls for the STK1p values. The mean age of the healthy controls was 46.1±10.5 years (range, 30–67 years).

### Survival

The patients were followed up for 44 months by tracking medical records, letter and telephone communication. Information on the survival status of the patients was obtained until May 5, 2011. No information was available for 13 patients. The followed-up patients included patients with advanced (n=2), locally advanced (n=34) and early locally advanced (n=2) tumors.

### Treatments

The neoadjuvant and the adjuvant chemotherapy were individual treatment programs, depending on the individual physiological and clinical symptoms. The treatment was designed according to the NCCN recommendations for breast cancer patients. The patients were treated with a combination of different types of drugs. Neoadjuvant therapy was administered for 1–4 cycles and adjuvant therapy for 1–6 cycles, depending on the clinical response, which was evaluated by imaging after each cycle. The types of drugs used for neoadjuvant therapy were as follows: T, 1 patient; TA, 1 patient; TAC, 7 patients; TEC, 2 patients; FAC, 21 patients; FEC, 7 patients; TEC+FEC, 1 patient; TAC+FEC, 1 patient; and TAC+FAC, 2 patients. Neoadjuvant treatment was not administered to 8 patients due to a small tumor burden. The drugs used for adjuvant therapy were as follows: T, 5 patients; TA, 1 patient; TAC, 5 patients; TEC, 2 patients; TP, 4 patients; FAC, 11 patients; FEC, 6 patients; T+AC, 4 patients; T+FAC, 10 patients; and T+FEC, 3 patients. The treatment cycle time was 21 days, with a 7-day rest between the cycles. The concentrations were adjusted according to the NCCN guidelines. For the drug abbreviations, see the NCCN guidelines.

### STK1 assay

The concentration of STK1 was measured by using a commercial kit based on an enhanced chemiluminescence (ECL) dot blot assay (SSTK Biotech Ltd.) (13,14). Samples comprising 3 μl of serum were directly applied onto nitrocellulose membranes. The serum samples were probed with anti-TK1 chicken IgY antibody raised against a peptide (residue 195–225, GQPAG PDNKE NCPVP GKPGE AVAAR KLFAPQ). The TK1 peptide was dotted at different concentrations (20, 6.6 and 2.2 pM) to obtain an extrapolation standard curve. The intensities of the spots on the membrane were determined by a charge-coupled device camera (CIS-I Imaging System, SSTK Biotech Ltd.). The results were analysed using a computer program provided by SSTK Biotech Ltd.

### Receiver operating characteristic (ROC) analysis

The ROC analysis of the STK1p results was performed on preoperative patients with early local breast cancer (n=120) compared to healthy Chinese female volunteers (n=286). The healthy controls were free of any known disease. The serum samples from the breast cancer patients were collected at the Department of Oncology, Karolinska University Hospital, Sweden. No difference in the STK1p values was observed between healthy Chinese and Swedish individuals.

### Western blot analysis

The western blot analysis of TK1 in the serum was performed as previously described (13).

### Statistical analysis

The Kaplan-Meier method and the log-rank test were used for the comparison of survival rates. Cox regression was used for the univariate and multivariate analyses. The Chi-square and Student’s t-tests were used for the comparison of TK1 expression among patients with different pathological stages, ER, PgR and HER2 status and CR to treatment. The ROC analysis was performed by a computer software program provided by Analyse-It Software, Leeds, UK. P<0.05 was considered to indicate a statistically significant difference.

### Study approval

The patients provided verbal consent to participate in this study. The study was approved by the Committee on Research Ethics at Xiangya Hospital, Zhongnan University, Changsha, China. This study was conducted in accordance with the Helsinki Declaration of 1983.

## Results

### Specificity of anti-TK1 antibody and sensitivity of STK1p assay

**Figure 1:**
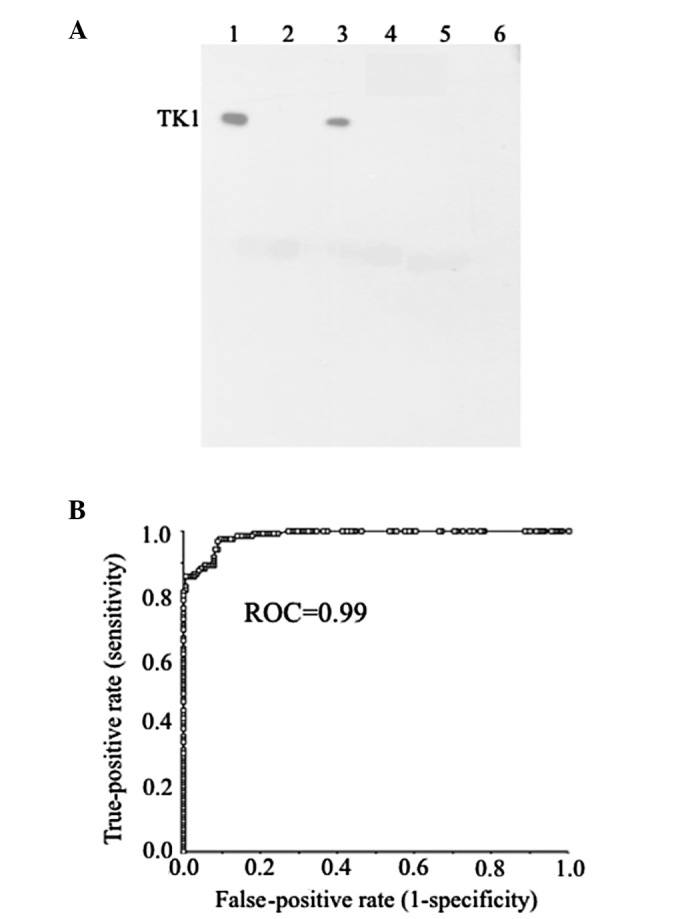
(A) Example of a western blot analysis of thymidine kinase 1 (TK1) in the serum of a patient with breast cancer (patient no. 8072). Lane 1, at the time of surgery; lane 2, sample buffer as control; lane 3: 6 months after surgery; lane 4: sample buffer as control; lanes 5–6, competing antigenic tests in the presence of 500 nM of a 31-amino acid peptide; lane 5: at the time of surgery; lane 6, 6 months after surgery. The weak bands below TK1 are from compounds present in the buffer. (B) ROC analysis of breast cancer patients (n=120) in relation to healthy individuals (n=286).

In order to assess the usefulness of the STK1p assay, the specificity and sensitivity of the ECL dot blot STK1p assay system were investigated by western blotting and ROC analysis. In the western blot analysis of native serum TK1 from breast cancer patients prior to treatment, only one band corresponding to human TK1 was observed, demonstrating the high specificity of the anti-TK1 IgY antibody (Fig. 1A). A competing experiment adding an excess of antigen (a 31-amino acid peptide, 500 nM) revealed no detectable bands, providing additional evidence for the high specificity of the IgY anti-TK1 antibody (Fig. 1A).

The ROC-analysis demonstrated that the sensitivity was high (Fig. 1B, Table II). At an optimized STK1p cut-off value of 2.00 pM, the sensitivity and specificity were 0.86 and 0.99, respectively. The area under the curve was 0.99 and the likelihood (+) value was 153.64. The high ROC and likelihood values demonstrated that the STK1p assay was able to efficiently distinguish between groups of healthy individuals and breast cancer patients.

### Patients

A total of 51 patients were recruited for this study. The majority of patients had stage II (54.9%) and III (31.4%) disease (Table I). There was a statistically significant higher frequency of ER−, PgR− and HER2-receptor negative patients [triple-negative breast cancer (TNBC)] that succumbed to the disease within 44 months after treatment, compared to ER+, PR+ and HER2− patients (Table I). Moreover, 72.5% (37/51) of the patients exhibited a PR upon treatment, whereas 5.9% (3/51) achieved a CR and others had SD 3.9 % (2/51) or PD 2% (1/51). Patients with a tumor size >5.0 cm exhibited a statistically significant shorter survival, compared to patients with a tumor size <5.0 cm (P=0.006, data not shown).

The STK1p levels were determined 1 week following the completion of the neoadjuvant treatment and prior to surgery, and at 1, 3 and 6 months after surgery. In this group of patients no pre-treatment STK1p serum samples were obtained due to the routine clinical procedure. The serum samples at 1 and 3 months after surgery were collected during the adjuvant chemotherapy and were handled with caution due to the uncontrolled fluctuations in the STK1p levels caused by chemotherapy-induced tumor cell disintegration. By contrast, the STK1p levels at 6 months after surgery were stabilized and mainly reflected the tumor burden. The serum samples prior to the initiation of the neoadjuvant treatment were only obtained from 11 patients due to the routine clinical procedure and, thus, were not included in the statistical evaluation of the results. At the end of neoadjuvant treatment, the average STK1p values of these 11 patients were decreased by 46.9%, indicating reduced proliferation rates of the breast tumors as a result of the neoadjuvant treatment (data not shown).

There was no significant difference in the mean STK1p levels after neoadjuvant treatment and at 1, 3 and 6 months after surgery (Table III), or in the number of patients with low or high STK1p values at these time points (Table IV). This is likely due to fluctuations in STK1p values caused by the chemotherapy, at least at 1 and 3 months after surgery. However, there was a statistically significant difference in the STK1p values between the low and high STK1p groups at 3 and 6 months after surgery (Table III).

**Figure 2:**
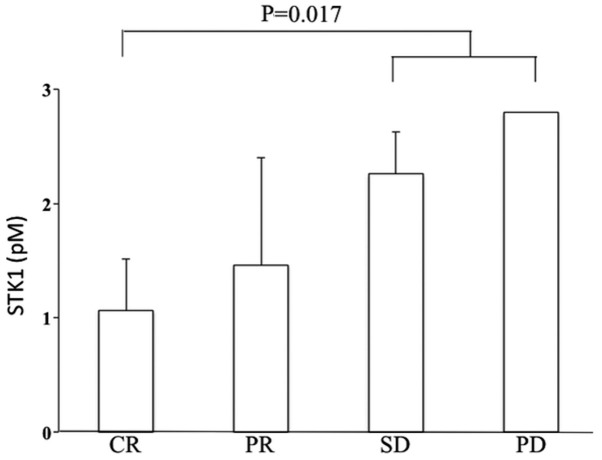
STK1p values in relation to clinical response at 6 months after surgery. CR, n=3; PR, n=37; SD, n=2; PD, n=1. STK1p, serum thymidine kinase 1 protein; CR, complete response; PR, partial response; SD, stable disease, PD, progressive disease.

Patients with SD and PD exhibited significantly higher STK1p values at 6 months after surgery, compared to patients with CR (Fig. 2). These differences were not observed in the serum samples from patients at 1 and 3 months after surgery (data not shown).

### STK1p values and metastatic disease

**Figure 3:**
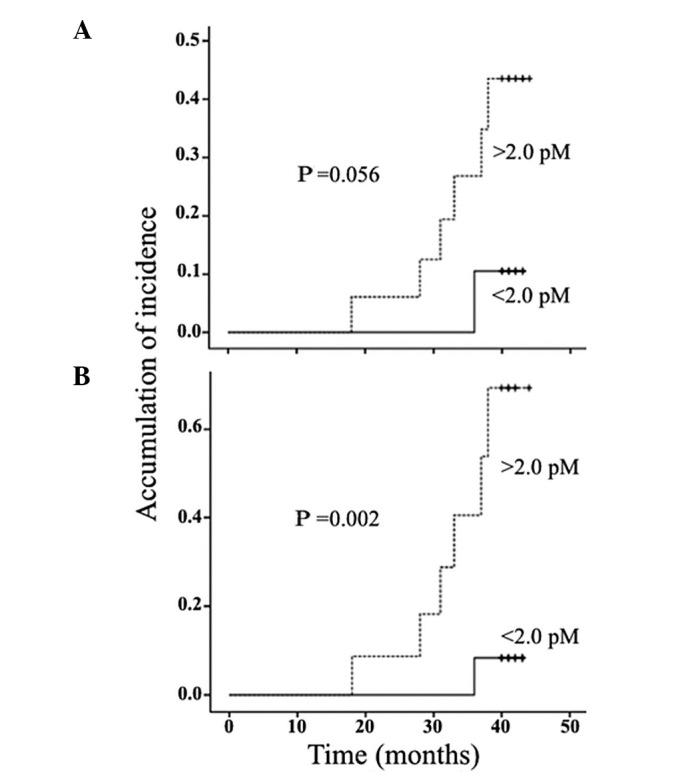
Serum thymidine kinase 1 levels in relation to the development of metastasis at (A) 3 months and (B) 6 months following surgery.

Patients with high STK1p values (≥2.0 pM) at 3 and 6 months after surgery (Fig. 3A and B) had a statistically significant higher risk of developing metastasis, starting at 18 months and continuing up to the end of the observation period, at 42 months.

A Cox univariate analysis of the variables 6 months after surgery demonstrated that STK1p levels, clinical stage and tumor size, but not age, were associated with the development of metastasis (Table V). A multivariate analysis at 6 months demonstrated that STK1p was the only independent prognostic marker for the occurrence of metastatic disease (Table V). High STK1p values (≥2.0 pM) 6 months after surgery were associated with an increased risk of metastasis by 8.2-fold (Table V).

### STK1p values and overall survival

**Figure 4:**
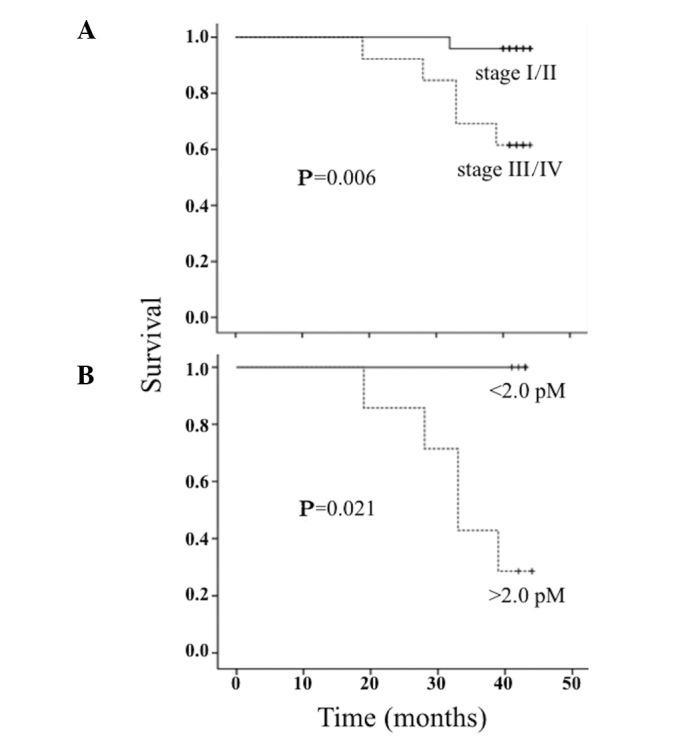
(A) Survival of patients with clinical stage I/II and III/IV disease. (B) Serum thymidine kinase 1 levels in relation to the survival of patients with stage III/IV disease.

**Figure 5:**
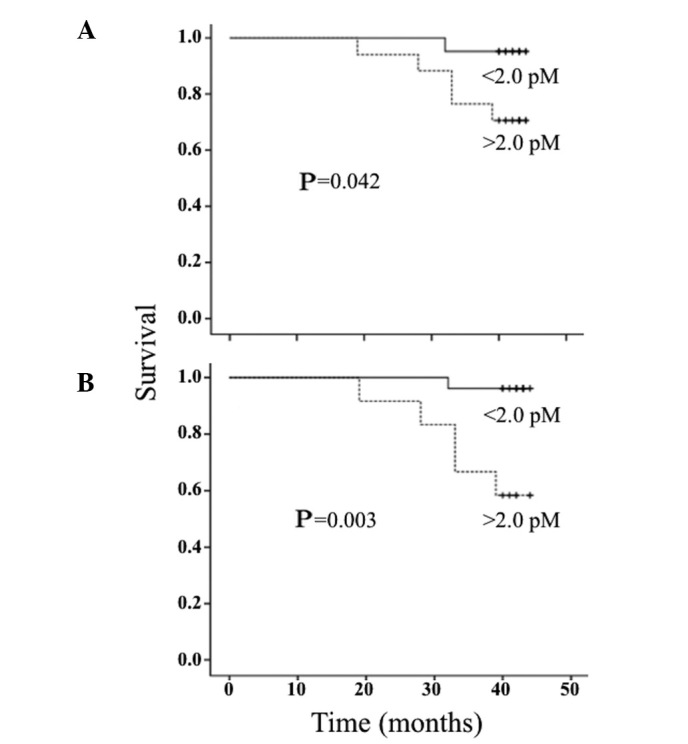
Serum thymidine kinase 1 levels in relation to survival at (A) 3 months and (B) 6 months following surgery.

There was a statistically significant difference in survival between patients with stage I/II and stage III/IV disease (Fig. 4A). Patients with high STK1p values (≥2.0 pM) exhibited a significantly shorter survival compared to patients with low STK1p values (<2.0 pM) at 3 and 6 months after surgery (Fig. 5A and B), but not in the serum samples obtained 1 month after surgery (data not shown). Of note, patients with stage III/IV disease and low STK1p values (<2.0 pM, 5/12) exhibited a longer survival compared to patients with high STK1p values (≥2.0 pM, 7/12) (Fig. 4B).

In a univariate analysis of patients at 3 and 6 months after surgery, STK1p levels, clinical stage and tumor size, but not age, were statistically significantly associated with survival (Table VI). In a multivariate analysis, STK1p was found to be the only independent prognostic factor for survival (Table VI). High STK1p values (≥2.0 pM) at 3 and 6 months after surgery increased the mortality risk by 11- and 12-fold, respectively (Table VI).

## Discussion

Breast cancer development and progression involves complex interactions between hormonal receptors and signaling pathways of growth factors, some of which are evident in the serum. In patients with locally advanced/advanced breast cancer, prognostic pathological markers such as nodal disease, presence of inflammatory breast cancer or poor pathological response and serum biomarkers such as carcinoembryonic antigen, CA15-3, MMP-2, MMP-9, tissue polypeptide antigen, tissue polypeptide-specific antigen, epidermal growth factor receptor and HER2/neu, have been investigated as predictors of clinical or pathological response to chemotherapy (15,16). However, the results of these investigations were not successful in identifying specific biomarkers for each chemotherapeutic regimen. Recently, the significance of the HER2 profile for the management and treatment of primary breast carcinoma was investigated. One study reported that ER and HER2 immunohistochemistry and HER2 fluorescence *in situ* hybridization were not significantly different in primary breast carcinomas prior to and following neoadjuvant chemotherapy (17). Another study reported that decreasing serum HER2/neu values were observed following neoadjuvant chemotherapy and were correlated with pathological response (18). However, Mazouni *et al*(19) did not observe any difference in HER2/neu levels between patients with pathological CR and those with residual disease. Furthermore, a summary of 65 studies on the association of HER2/neu with prognosis in breast cancer patients did not lead to any definitive conclusions (20). Receptor CXCR4 was overexpressed in HER2-negative breast cancer patients and was correlated with disease-free survival. This was not the case in HER2-positive breast cancer patients, suggesting that receptor CXCR4 may be used to distinguish between patients with long- and short-term survival (21). In a previous study, we demonstrated that HER2 overexpression was associated with significantly higher STK1p values prior to neoadjuvant chemotherapy (22). In addition, TNBC patients (ER−, PgR− and HER2−) have poor prognostic characteristics compared to other subtypes, due to lack of common therapeutic targets (23–26). TNBC patients with residual disease following neoadjuvant chemotherapy exhibit significantly worse survival compared to non-TNBC patients, particularly during the first 3 years after treatment (23). In the present study, despite the limited patient sample, TNBC was found at a significantly higher frequency among patients who succumbed to metastatic disease, compared to patients with ER+, PR+ and HER2−, confirming recent studies (8,14). Despite the vast availability of prognostic markers, the results remain confusing and there is need of alternative biomarkers for monitoring the treatment of breast cancer patients.

To the best of our knowledge, this study was the first to demonstrate that the concentration of STK1p is a reliable monitoring and prognostic marker for the treatment outcome of patients with advanced breast cancer. The STK1p levels are able to predict the development of metastasis at 6 months after surgery, i.e., 12 months prior to the first patients being diagnosed with metastasis. This was in accordance with findings of a previous study on low-risk breast cancer patients, in which the STK1p levels predicted recurrence at 6 months after surgery (27). STK1p is also a more reliable prognostic factor for overall survival compared to other parameters such as clinical stage, tumor size and age. Based on a multivariate Cox analysis, STK1p was found to be an independent predictive marker for the development of metastasis and a prognostic marker for survival as early as 3–6 months after surgery. The efficacy of STK1p as a prognostic marker was even more apparent in the serum samples collected at 6 months after surgery. Of note, in this study the STK1p levels were able to identify a group of high-stage (III/IV) patients with good prognosis, although the follow-up time was <5 years. Patients with stage III/IV disease are considered as exhibiting poor survival, which was also demonstrated by this study (Fig. 5A). Similar results were reported by a study on pT1 lung carcinoma patients (28), in which TK1 expression, as determined by immunohistochemistry of lung tumor tissues, was correlated with survival. Data concerning the expression of TK1 in tissues obtained from patients with cervical carcinoma also confirm these results (29)

TK1 levels in the serum may be determined by the enzyme activity or concentration. STK1p was used in this study, since it was previously demonstrated that STK1p levels in patients with solid tumors provide more accurate prognostic information in a larger proportion of the patients, compared to STK1a (8), as the sensitivity of STK1 activity detection methods is usually low in patients with solid tumors. This was also the case in a recent study by Nisman *et al*(30) which compared tissues from primary breast cancer patients with benign breast tissues. The conclusions of that study were that the Liaison TK assay and the highly sensitive DiviTum TK activity assay may be used to predict disease recurrence in the preoperative setting. However, the sensitivity for the two assays was ~25% and there was no significant difference in STK1a levels between the benign and malignant breast disease groups. However, several previous studies have reported a sensitivity of the STK1p assay of >80% (22,31,32). In the present study, using an optimized threshold cut-off STK1p value of 2.0 pM, high ROC value (0.99), high sensitivity (0.86) and high specificity (0.99) were observed, supporting the use of the STK1p assay over that of STK1a assays in breast cancer patients. Thus, STK1p determination provides more accurate predictive and prognostic information regarding recurrence and survival compared to enzyme activity measurements. A possible explanation for the discrepancy between STK1p and STK1a assays is that only a subset of the TK1 molecules in the serum is enzymatically active. Inactivation of TK1 may be the result of tumor cell death leading to denaturation/inactivation of the enzyme.

Depending on the cut-off value, different levels of sensitivity and specificity were obtained. In our meta-study on health screening in 2011 (including a total of 35,365 participants) (30), we optimized the STK1p cut-off value in order to limit the number of false-positive cases. At a cut-off value of 2.00 pM, the specificity and sensitivity were 0.99 and 0.80, respectively. The ROC and likelihood values were high (0.96 and 233.73, respectively). The ROC analysis was performed on serum samples obtained from 720 cancer patients with 11 different types of tumors and 4,103 sub-healthy individuals without known malignancies or premalignant conditions. The ROC values of the different types of tumors were 0.92–1.00. Thus, the STK1p assay appears to be adequately sensitive for health screening and for use in clinical oncology.

In conclusion, this study was based on patients following routine individual treatment and, thus, is not considered a clinical trial. However, significant differences were observed between the different groups investigated, suggesting that STK1p is a useful prognostic and predictive biomarker for monitoring treatment, the development of metastasis and survival in the routine clinical setting. Similar studies on STK1p levels in other types of malignancies support this conclusion (8,14). In addition, STK1p may be able to identify late-stage (III/IV) breast cancer patients with better survival expectancy, leading to improved personalized treatment. However, in order to obtain a more complete clinical evaluation of the usefulness of STK1p in breast cancer management, a larger case-control clinical trial is required and is currently in progress.

## Figures and Tables

**Table I:** Patient characteristics.

Characteristics	No.	P-value
Patients	51	
Mean age (years) (range)	45.7±8.7 (30–67)	
Follow-up (months)	44	
Pre-neoadjuvant stage		
I	3	
II	28	
III	16	
IV	4	
ER, PgR, HER2 status		
ER+, PgR+, HER2−, total	11	
Death from metastasis	0	
ER−, PgR−, HER2− (TNBC), total	7	A vs. B: 0.049
Death from metastasis	3	
ER−, PgR−, HER2+, total	12	A vs. C: 0.191
Death from metastasis	2	
Other subtypes, total	8	A vs. D: 0.256
Death from metastasis	1	
Tumor size (cm)		
<5.0	36	
>5.0	15	
Clinical response		
CR	3	
PR	37	
SD	2	
PD	1	

Statistical analysis was performed by the Chi-square test. ER, estrogen receptor; PgR, progesterone receptor; HER2, human epidermal growth factor receptor 2; TNBC, triple-negative breast cancer; CR, complete response; PR, partial response; SD, stable disease, PD, progressive disease. A, ER+, PR+, HER-2−, all; B, ER−, PR−, HER-2− (TNBC), all; C, ER−, PR−, HER-2+, all; D, other subtypes, all.

**Table II:** ROC analysis of breast cancer patients (n=120) in relation to healthy individuals (n=286).

Type	ROC value	SE	Z	P-value	Sensitivity	Specificity	Likelihood (+)	n
Breast cancer patients	0.99	0.004	110.95	<0.0001	0.86	0.99	153.64	120

ROC, receiver operating characteristic; SE, standard error.

**Table III:** STK1p values during treatment in the low (<2.0 pM) and high (≥2.0 pM) STK1p groups.

Time point	STK1p (pM)	Low	High	P-value
Healthy controls	0.5±0.4	-	-	
1 week^a^	1.8±0.9	-	-	
1 month^a^	1.6±1.1	-	-	
3 months^a^	1.8±1.1	1.2±0.5	3.0±1.0	
6 months^a^	1.4±0.9	1.1±0.6	2.8±0.6	<0.001

a After neoadjuvant treatment.

Values are presented as means ± standard deviation. Statistical analysis was performed using the Student’s t-test. STK1p, serum thymidine kinase 1 protein.

**Table IV:** Number of patients with low (<2.0 pM) and high (≥2.0 pM) STK1p values during treatment.

Time point	Low	High
1 week^a^	34	17
1 month^a^	35	16
3 months^a^	33	18
6 months^a^	38	13

a Following neoadjuvant treatment.

STK1p, serum thymidine kinase 1 protein.

**Table V:** Cox values of variables in relation to risk of metastasis at 3 and 6 months following surgery.

Variables	P-value	Hazard risk	95% CI
3 months			
Univariate			
STK1p (<2.0 vs. ≥2.0 pM)	0.057	-	-
Stage (I/II vs. III/IV)	0.017	7.077	1.43–35.16
Size (<5.0 vs. ≥5.0 cm)	0.017	7.077	1.43–35.16
Age (<50 vs. ≥51 years)	0.950	-	-
Multivariate			
STK1p (<2.0 vs. ≥2.0 pM)	0.548	-	-
Stage (I/II vs. III/IV)	0.017	7.077	1.43–35.16
Size (<5.0 vs. ≥5.0 cm)	0.511	-	-
Age (<50 vs. ≥51 years)	0.898	-	-
6 months			
Univariate			
STK1p (<2.0 vs. ≥2.0 pM)	0.010	8.221	1.67–40.87
Stage (I/II vs. III/IV)	0.017	7.077	1.43–35.16
Size (<5.0 vs. ≥5.0 cm)	0.017	7.077	1.43–35.16
Age (<50 vs. ≥51 years)	0.950	-	-
Multivariate			
STK1p (<2.0 vs. ≥2.0 pM)	0.010	8.221	1.67–40.87
Stage (I/II vs. III/IV)	0.087	-	-
Size (<5.0 vs. ≥5.0 cm)	0.087	-	-
Age (<50 vs. ≥51 years)	0.312	-	-

CI, confidence interval; STK1p, serum thymidine kinase 1 protein.

**Table VI:** Cox values of variables in relation to survival at 3 and 6 months following surgery.

Variables	P-value	Hazard risk	95% CI
3 months			
Univariate			
STK1p (<2.0 vs. ≥2.0 pM)	0.080	-	-
Stage (I/II vs. III/IV)	0.028	11.071	1.29–94.92
Size (<5.0 vs. ≥5.0 cm)	0.028	11.071	1.29–94.92
Age (<50 vs. ≥51 years)	0.891	-	-
Multivariate			
STK1p (<2.0 vs. ≥2.0 pM)	0.028	11.071	1.29–94.92
Stage (I/II vs. III/IV)	0.493	-	-
Size (<5.0 vs. ≥5.0 cm)	0.540	-	-
Age (<50 vs. ≥51 years)	0.940	-	-
6 months			
Univariate			
STK1p (<2.0 vs. ≥2.0 pM)	0.021	12.67	1.48–108.79
Stage (I/II vs. III/IV)	0.028	11.07	1.29–94.92
Size (<5.0 vs. ≥5.0 cm)	0.028	11.07	1.29–94.92
Age (<50 vs. ≥51 years)	0.891	-	-
Multivariate			
STK1p (<2.0 vs. ≥2.0 pM)	0.010	12.67	1.65–40.87
Stage (I/II vs. III/IV)	0.497	-	-
Size (<5.0 vs. ≥5.0 cm)	0.122	-	-
Age (<50 vs. ≥51 years)	0.122	-	-

CI, confidence interval; STK1p, serum thymidine kinase 1 protein.
